# Mitral-Aortic Intervalvular Fibrosa Pseudoaneurysm: A Rare and Life-Threatening Presentation

**DOI:** 10.7759/cureus.108948

**Published:** 2026-05-16

**Authors:** Gayathry Rajasekharan, Chinthu Sara Jacob, Azad Shams, Ajithkumar Sivasankaran, Parvathy Rajasekharan, Rajasekharan Chandrasekharan

**Affiliations:** 1 Pathology, Government Medical College, Thrissur, IND; 2 General Medicine, Sree Uthradom Thirunal Academy of Medical Sciences, Thiruvananthapuram, IND; 3 Internal Medicine, Azeezia Institute of Medical Science and Research, Kollam, IND; 4 Oncology, PRS Hospital, Thiruvananthapuram, IND

**Keywords:** aortic root abscess, aorto-cavitary fistula, infective endocarditis, mitral-aortic intervalvular fibrosa, pseudoaneurysm

## Abstract

A 40-year-old male with no prior history of cardiac disease or other comorbidities presented to the emergency department with a history of high-grade intermittent fever and breathlessness for two weeks. Examination revealed a febrile patient who had features of mitral regurgitation and aortic regurgitation. A transthoracic echocardiogram (TTE) revealed vegetation in the anterior mitral leaflet, along with moderate aortic and mitral regurgitation and a ruptured aortic root abscess that formed dual fistulous tracts in the aortic root. An extremely uncommon complication of infective endocarditis affecting the aortic valve is the development of an abscess in the intervalvular fibrosa (IVF) dual fistula formation. A dual fistulous tract communicating with the aortic root and the ascending aorta is very sparsely described in the literature. A 64-slice computed coronary angiography confirmed a pseudoaneurysm posterior to the aortic root, communicating with the aortic root through a neck and with the left ventricular outflow tract.

## Introduction

The aortic root forms the central part of the heart and is of particular significance, as it maintains anatomical and functional relationships with all cardiac chambers through its walls. It comprises the aortic valve leaflets; the associated sinuses (sinuses of Valsalva); the sinotubular junction (STJ), which marks the transition to the ascending aorta; and the ventriculo-aortic junction (VAJ), which represents the upper boundary of the left ventricular outflow tract (LVOT) [1].

The aortomitral curtain, a relatively avascular fibrous structure, is particularly vulnerable to the spread of infection. Periannular extension of infection can result in serious complications such as heart failure, ventricular septal defects, conduction abnormalities, including heart block, and aortico-cavitary fistulae, the latter occurring in approximately 1.5% to 2.2% of cases. Prompt and accurate diagnosis of native aortic valve endocarditis is crucial for effective management and prevention of these complications [2].

The mitral-aortic intervalvular fibrosa (MAIVF), which provides structural continuity between the aortic and mitral valves, is predisposed to injury and infection due to its avascular nature and thin structure. Aortic valve endocarditis may extend to involve the MAIVF and anterior mitral leaflet either through direct spread or via the impact of an infected aortic regurgitant jet. Untreated MAIVF abscesses may progress to pseudoaneurysm formation, eventually rupturing into adjacent cardiac chambers and resulting in aorto-cavitary fistulae [3].

Pseudoaneurysm of the intervalvular fibrosa (PIVF), particularly when associated with dual fistulous communication, is an uncommon but serious condition. It is most often associated with infective endocarditis but may also occur following cardiac surgery, trauma, or degenerative processes. Involvement of the MAIVF is associated with significant morbidity and mortality [4]. Here, we report an unusual case of infective endocarditis complicated by an aortic root abscess with subsequent pseudoaneurysm formation and dual fistulous communication with the LVOT.

## Case presentation

A 40-year-old male, who is a manual laborer, presented with complaints of high-grade, intermittent fever for two weeks associated with breathlessness. The patient had baseline dyspnea of New York Heart Association (NYHA) class II for one week, worsening to class IV over the preceding two weeks. There was no history of chest discomfort, palpitations, cough, expectoration, or pedal edema. There was no prior history of rheumatic fever, diabetes mellitus, hypertension, or known structural heart disease. He was a non-smoker and did not consume alcohol. There were no identifiable risk factors for coronary artery disease or a history of intravenous drug abuse.

On examination, the patient was dyspneic at rest. Pulse rate was 100/min, blood pressure was 140/60 mmHg (right upper limb), temperature was 100.4°F, and respiratory rate was 18/min. He was pale and had bilateral pitting pedal edema. Cardiovascular examination revealed a just-visible jugular venous pulse at the root of the neck. The apical impulse was located in the sixth intercostal space, 2 cm lateral to the midclavicular line. No palpable thrill was noted. On auscultation, an early diastolic murmur (grade 3/6) was heard in the aortic area, accentuated on expiration, along with a pansystolic murmur (grade 3/6) at the apex. No peripheral stigmata of infective endocarditis were observed. Respiratory examination revealed bilateral basal crepitations. Abdominal and central nervous system examinations were unremarkable.

Initial laboratory evaluation revealed anemia and leukocytosis with a marked neutrophilia. There was a significant increase in inflammatory markers. Renal and liver function tests were within normal limits, and a vasculitis workup was negative. Blood cultures remained sterile. Given the clinical suspicion, an extensive workup for culture-negative endocarditis was performed, including blood cultures and targeted serological investigations for atypical organisms; all results were negative. Results of quantitative investigations, details, and the corresponding reference ranges are summarized in Table 1.

**Table 1:** Quantitative investigation details and the corresponding reference ranges Quantitative laboratory findings at the time of admission. Results highlight a systemic inflammatory response, evidenced by significant leucocytosis, neutrophilia, and elevated acute-phase reactants (ESR, CRP, and procalcitonin). ESR: Erythrocyte sedimentation rate, CRP: C-reactive protein

Investigation	Quantitative value	Reference range	Result attribute
Hemoglobin	9.2 g/dL	12.0-16.0 g/dL	Decreased
Total leukocyte count	16,400/mm³	4,000-11,000/mm³	Increased
Neutrophils	88%	40%-75%	Increased
Lymphocytes	10%	20%-45%	Decreased
Eosinophils	2%	1%-4%	normal
Erythrocyte sedimentation rate (ESR)	76 mm/hour	0-20mm/hour	Increased
C-reactive protein (CRP)	70 mg/L	< 10 mg/L	Increased
Serum procalcitonin	2 ng/mL	< 0.5 ng/mL	Increased

**Figure 1:**
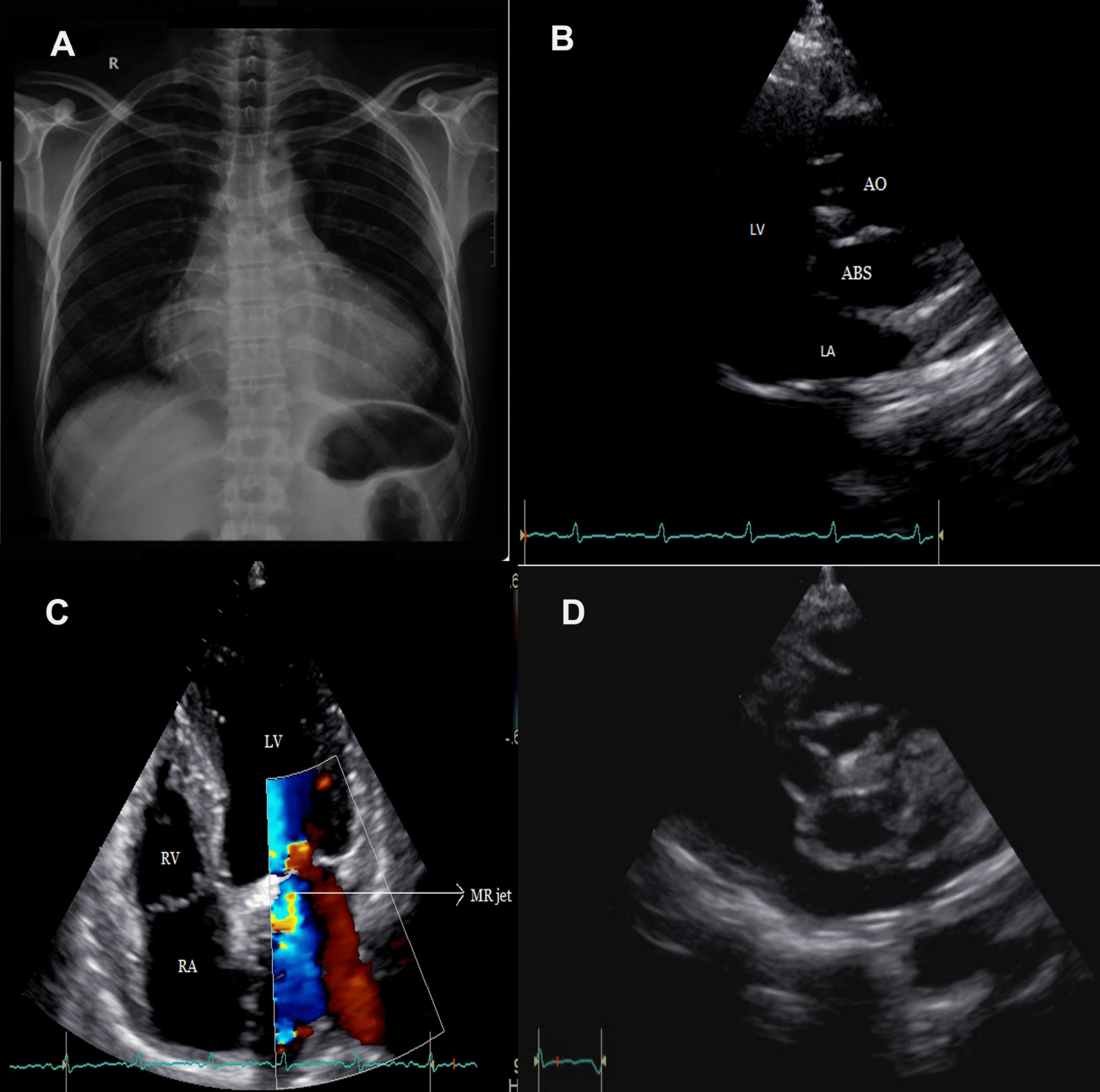
Chest radiograph showing cardiomegaly The TTE parasternal long-axis view shows an echo-lucent space (denoted by 'ABS') adjoining the area of MAIVF (A), and this space lies posterior to the aorta, bulging into the left atrium (B). The apical four-chamber view shows the jet of mitral regurgitation (C). The parasternal short-axis view reveals the bicuspid aortic valve (D). MAIVF: Mitral-aortic intervalvular fibrosa, TTE: Transthoracic echocardiogram, LV: Left ventricle, RV: Right ventricle, RA: Right atrium, LA: Left atiruma, MR: Mitral regurgitation

The ECG revealed a sinus rhythm at 100 beats/min with a normal PR interval. Chest radiograph showed cardiomegaly (Figure 1A). A transthoracic echocardiogram (TTE), parasternal long-axis view, showed an echo\lucent space adjoining the area of the MAIVF, and this space lies posterior to the aorta, bulging into the left atrium (Figure 1B). The apical four-chamber view showed the jet of mitral regurgitation (Figure 1C). The parasternal short-axis view showed a bicuspid aortic valve (Figure 1D). 

Videos of the apical four-chamber view demonstrated the vegetation attached to the anterior mitral leaflet and mitral regurgitation (Video 1A). The parasternal long-axis view helped visualize the aortic regurgitation and mitral regurgitation jets. An echolucent chamber was seen adjacent to the MAIVF lying posterior to the aorta (Video 1B). The Doppler demonstrated the flow of blood from the aorta into the MAIVF aneurysm, which fills in systole and empties in diastole (Video 1C and Video 2).

**Video 1:** Apical four-chamber view revealing the vegetation attached to anterior mitral leaflet and mitral regurgitation A: Parasternal long-axis view revealing the aortic regurgitation and mitral regurgitation jet; B: Echolucent chamber adjacent to the MAIVF that lies posterior to the aorta; C: Doppler revealing flow of blood from the aorta into the MAIVF aneurysm, which fills in systole and empties in diastole. MAIVF: Mitral-aortic intervalvular fibrosa

**Video 2:** Doppler demonstrating the flow of blood from the aorta into the MAIVF aneurysm that fills in systole and empties in diastole MAIVF: Mitral-aortic intervalvular fibrosa

**Figure 2:**
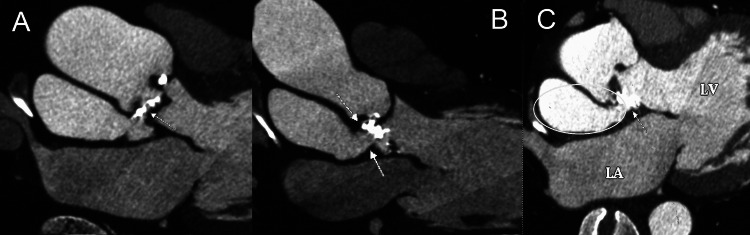
CT coronary angiography A: Blood-filled cavity posterior to the aortic root communicating with the aortic root via a neck measuring 4.3 mm and a heavily calcified aortic valve (arrow); B: Another blood-filled cavity noted posterior to the aortic root communicating with the LVOT through a neck measuring 8 mm; C: Blood-filled cavity of size 5.3 x 5.4 x 2.3 mm posterior to the aortic root and ascending aorta. Posteriorly, the cavity closely abuts the left atrium without any significant compression, with no communication noted with the left atrium. LVOT: Left ventricular outflow tract

A CT coronary angiography revealed a blood-filled cavity noted posterior to the aortic root, communicating with the aortic root through a neck measuring 4.3 mm. The aortic valve was found to be heavily calcified (Figure 2A). Another blood-filled cavity was noted posterior to the aortic root, communicating with the LVOT through a neck measuring 8 mm (Figure 2B). A blood-filled cavity of size 5.3 x 5.4 x 2.3 mm was noted posterior to the aortic root and ascending aorta. Posteriorly, the cavity was closely abutting the left atrium without any significant compression. No communication was noted with the left atrium (Figure 2C). 

A diagnosis of pseudoaneurysm of MAIVF with an aortic root abscess and dual fistula was made. The patient was started on intravenous ceftriaxone and vancomycin along with supportive therapy. He underwent an emergency modified Bentall procedure with aortic root replacement.

Intraoperatively, the aneurysm was found to extend around the aortic root with communication to the left and non-coronary sinuses and extension to the aortomitral continuity. The postoperative period was uneventful. Upon review in the first, second, and fifth years, he was found to be hemodynamically stable.

## Discussion

The presence of MR, a bicuspid aortic valve, and infective endocarditis reflects a complex cardiovascular disease process that culminated in severe complications. A bicuspid aortic valve, a congenital malformation affecting 1% to 2% of the population, has a 12-fold higher risk of infective endocarditis(preferentially native valve) compared to the general population. Altered flow patterns through a bicuspid aortic valve may contribute to increased endothelial damage and subsequent platelet and fibrinogen deposition, which can facilitate the seeding of hematogenous bacteria or fungi. Patients with a bicuspid aortic valve more frequently experience isolated aortic valve infective endocarditis, suggesting that this may be a preferential site for bacterial growth [5]. Patients with a bicuspid aortic valve and IE incur a high risk of abscess formation and require early surgery in almost three-quarters of cases [6].

The mechanism of development of infective endocarditis in a bicuspid aortic valve is multifactorial. The turbulent blood flow, which can impose shearing stress and endothelial damage, creates a nidus for bacterial colonization; valve abnormalities predisposing to stasis, microthrombi formation, and increased risk of bacterial adherence and colonization; and endothelial dysfunction, which can increase platelet activation and adhesion, creating a favorable environment for bacterial colonization. Increased risk of bacteremia during dental procedures or infections at distant sites puts them at risk of infective endocarditis. Genetic predisposition may be another factor making some patients with a bicuspid aortic valve have increased susceptibility for infective endocarditis [5,7].

Mitral valve aortic endocarditis can occur through several mechanisms. A jet lesion resulting from turbulent flow from an infected aortic valve can hit the mitral valve, causing endothelial damage and risk of infection. A direct extension, in which infection can spread directly from the aortic valve to the mitral valve, or an extension of infection that involves aortic root or intervalvular fibrosa (IVF) seeding, can aid bacteria in colonizing and embolizing the mitral valve due to its anatomical proximity. Lastly, the aortic valve and mitral valve are in close proximity, which can facilitate the spread of the infection [8].

Culture-negative endocarditis (CNE) is a form of infective endocarditis in which routine blood cultures fail to identify a pathogen, occurring in about 5% to 20% of cases and complicating diagnosis and treatment. The most common cause is prior antibiotic exposure, which suppresses bacterial growth, while fastidious organisms (e.g., *Coxiella burnetii*; *Bartonella*; *Tropheryma whipplei*; *Haemophilus* species, *Aggregatibacter actinomycetemcomitans*, *Cardiobacterium hominis*, *Eikenella corrodens*, *Kingella kingae* (HACEK) group; and fungi) and laboratory limitations also contribute. Diagnosis requires a systematic approach, including repeated blood cultures, serology, PCR-based molecular methods, and echocardiography. Valve tissue analysis may aid diagnosis when surgery is performed. Management is primarily empirical with broad-spectrum antibiotics, later tailored to identified pathogens, and may require surgery in complicated cases. Overall, CNE demands a high index of suspicion and a multidisciplinary approach to improve outcomes [2].

The MAIVF is a delicate layer of avascular tissue, which is susceptible to injury and infection owing to its avascularity and thin nature (approximately 6 mm) [9]. Aortic valve endocarditis may culminate in infection of the MAIVF and the anterior mitral leaflet via direct infectious extension or impingement from an infected aortic regurgitant jet. In the absence of intervention, MAIVF infection can progress to abscesses, which could be complicated by pseudoaneurysm formation, with subsequent rupture into intracardiac chambers and the development of aorto-cavitary fistulae [10-13]. The occurrence of perivalvular abscess was significantly more frequent in patients with a bicuspid aortic valve (50% vs. 20%), which also correlated with a higher likelihood of requiring early surgery (82% vs. 57%) [5,9]. The role of predisposing risk factors has been recently evaluated, and current guidelines identify three classes of populations at risk: high risk, intermediate risk, and low risk. Patients with bicuspid aortic valves are included under intermediate-risk individuals [14].

Perivalvular extension is a complication of infective endocarditis that includes periannular or intramyocardial abscesses, mycotic pseudoaneurysms, and fistulae with a prevalence of 10% to 30% in native valve infective endocarditis and 30% to 55% in prosthetic valve endocarditis (PVE). The direct spread of infection to the valve annulus is common in infective endocarditis because of its relatively poor blood supply, resulting in diminished defense. Aortic root abscesses, representing a form of perivalvular extension, may result in persistent infection, heart failure, conduction abnormalities, and the formation of fistulous communications [10].

In our patient, the infective endocarditis appears to have originated at the level of the aortic valve. Bacterial colonization and subsequent inflammation can lead to leaflet destruction, severe aortic regurgitation, and paravalvular complications. These abscesses may remain localized or rupture into surrounding cardiac structures, forming ruptured aortic root abscess, pseudoaneurysm, and fistula formation [15].

In our patient, the aortic root abscess had ruptured, forming a dual fistula to the aortic root and the LVOT. The fistula created an abnormal communication between high-pressure and low-pressure cardiac chambers or vascular structures, resulting in continuous flow shunts, which is a life-threatening complication. Hemodynamically, this can lead to severe volume overload of the left ventricle and profound circulatory compromise. Fistulas from the aorta to the LVOT result in a continuous flow of blood from the aorta back into the LV during both systole and diastole. This exacerbates aortic regurgitation and can mask or worsen mitral valve regurgitation due to elevated LV pressures and dilatation.

Echocardiography remains the first level of imaging with either a transesophageal echocardiogram (TEE) or TTE, but it is strongly advised to have bimodality imaging, including a CT, nuclear imaging, or an MRI, to confirm or better characterize the cardiac lesions [14,16]. Compared to TEE, TTE has a low sensitivity and good specificity when evaluating infective endocarditis [14]. The TEE, while providing valuable information, is an invasive procedure with potential complications [17]. The CT offers a better approach for diagnosing infective endocarditis and its local complications, demonstrating superior detection of abscesses and pseudoaneurysms compared to echocardiography alone [18]. Echocardiography is superior in diagnosing vegetations, valvular leaflet perforations, and perivalvular leaks; meanwhile, CT is a useful tool when echocardiography is indeterminate and is a better tool for the detection of abscesses and pseudoaneurysms. Combining these two modalities can increase the sensitivity of diagnosing abscess/pseudoaneurysm up to 100% [18]. Notably, cardiac CT exhibits a 96% sensitivity in detecting valvular vegetations, a rate identical to that of multiplane TEE when benchmarked against surgical outcomes [14].

Medical management includes intravenous antibiotics tailored to the causative organism. Empirical therapy should cover staphylococci, streptococci, and enterococci. Early PVE or healthcare-associated infective endocarditis regimens should cover methicillin-resistant staphylococci and enterococci. Empiric antibiotics in community-acquired native valve endocarditis (NVE) or late PVE may include ampicillin with ceftriaxone or flucloxacillin and gentamicin as per the European Society of Cardiology (ESC) 2023 guidelines [19]. Emerging problems with antibiotic therapy include resistance and tolerance. Targeted therapy should follow identification of the pathogen. Treatment duration is typically four to six weeks, longer in PVE. Partial oral therapy may be considered in stable patients as per the POET (Partial Oral versus Intravenous Antibiotic Treatment of Endocarditis) trial findings [20].

Infective endocarditis may require surgical intervention, which offers a survival benefit of up to 20%. Indications include heart failure, uncontrolled infection, and prevention of embolization. Surgical techniques include the Bentall procedure, homograft, and complex reconstructions like the Commando procedure. The ESC 2023 guidelines emphasize a multidisciplinary heart team approach involving cardiology, cardiac surgery, infectious diseases, and imaging specialists to improve outcomes [14].

## Conclusions

Aortic root abscess and pseudoaneurysm formation are life-threatening complications of infective endocarditis and should be detected and treated at the earliest. Aortic valve endocarditis that does not respond to appropriate antibiotic therapy occurs not just in those with prosthetic valves but also with native valve involvement, like in bicuspid aortic valves. Persistent or recurring fever, elevated WBC, high CRP levels, new skin lesions, embolic events during treatment, progressively lengthening PR interval, or new onset of heart block on ECG should raise suspicion for this potentially fatal condition. In cases with a high index of suspicion, it will be prudent to take a TEE or TTE with a CT angiogram to increase the sensitivity to 100% for detection.
